# Fingerprint Systems: Sensors, Image Acquisition, Interoperability and Challenges

**DOI:** 10.3390/s23146591

**Published:** 2023-07-21

**Authors:** Akmal Jahan Mohamed Abdul Cader, Jasmine Banks, Vinod Chandran

**Affiliations:** 1 School of Electrical Engineering and Robotics, Queensland University of Technology, Brisbane 4000, Australia; j.banks@qut.edu.au (J.B.); vinod.chandran2@bigpond.com (V.C.); 2 Department of Computer Science, South Eastern University of Sri Lanka, Sammanthurai 32200, Sri Lanka

**Keywords:** finger biometrics, sensor, image acquisition, ultrasonic sensing, interoperability

## Abstract

The fingerprint is a widely adopted biometric trait in forensic and civil applications. Fingerprint biometric systems have been investigated using contact prints and latent and contactless images which range from low to high resolution. While the imaging techniques are advancing with sensor variations, the input fingerprint images also vary. A general fingerprint recognition pipeline consists of a sensor module to acquire images, followed by feature representation, matching and decision modules. In the sensor module, the image quality of the biometric traits significantly affects the biometric system’s accuracy and performance. Imaging modality, such as contact and contactless, plays a key role in poor image quality, and therefore, paying attention to imaging modality is important to obtain better performance. Further, underlying physical principles and the working of the sensor can lead to their own forms of distortions during acquisition. There are certain challenges in each module of the fingerprint recognition pipeline, particularly sensors, image acquisition and feature representation. Present reviews in fingerprint systems only analyze the imaging techniques in fingerprint sensing that have existed for a decade. However, the latest emerging trends and recent advances in fingerprint sensing, image acquisition and their challenges have been left behind. Since the present reviews are either obsolete or restricted to a particular subset of the fingerprint systems, this work comprehensively analyzes the state of the art in the field of contact-based, contactless 2D and 3D fingerprint systems and their challenges in the aspects of sensors, image acquisition and interoperability. It outlines the open issues and challenges encountered in fingerprint systems, such as fingerprint performance, environmental factors, acceptability and interoperability, and alternate directions are proposed for a better fingerprint system.

## 1. Introduction

Biometric systems are pervasive in people’s lives and assist to authenticate their identity reliably in many applications. Automated processes such as identification or verification are involved in a biometric recognition system where physical or behavioral characteristics of a biometric trait are used. Fingerprint identification is one of the most authentic approaches for human identification [1], where ridges and minutiae (ridge ending and branch) of the fingerprint information play a significant role in the recognition process [2,3,4,5,6,7]. The fingerprint is the oldest and a widely adopted biometric trait in forensic and civilian applications [8]. Until the use of DNA profiling, fingerprints were the central identification tool in criminal investigation. Later, it was widely used in government-related verification, for instance, integration of finger and face in passport and border control systems [9]. The fingerprint as a biometric modality is now prevalent in multiple applications related to civil activities such as attendance systems, access control, cellular authentication, e-commerce and information security applications [6,10].

**Figure 1:**
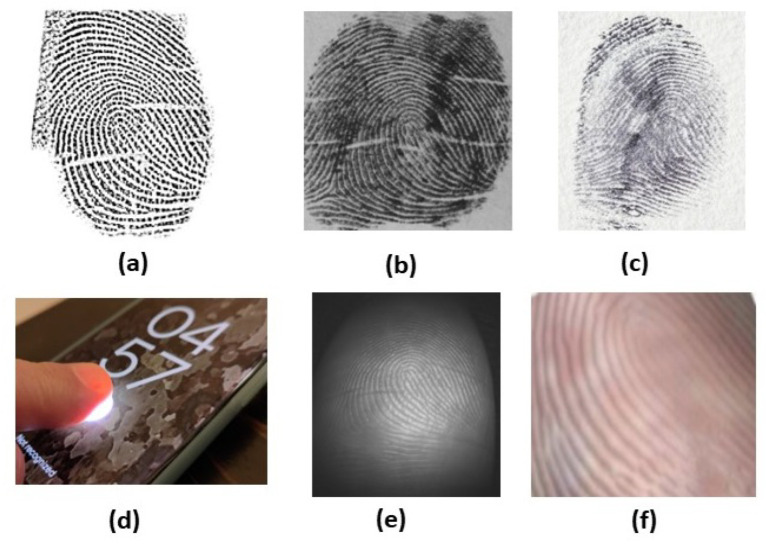
Variations of conventional contact and contactless fingerprints: (**a**) plain [22]; (**b**) rolled [22]; (**c**) latent [23]; (**d**) partial print [24]; (**e**) contactless 2D [22] and (**f**) contactless 3D. Reprinted with permission from Ref. [22], 2018, IEEE.

The fingerprint biometric system has been investigated using contact prints and latent and contactless images ranging from low to high resolution [2]. Contact-based fingerprint scanning systems occupy the larger portion of the state-of-the-art fingerprint recognition in civilian applications, while the contactless domain become attractive due to the presence of portable, compact and high-resolution cameras with different image capture strategies such as multispectral, multiview and 3D-image capture. While the imaging techniques are advancing with sensor variations, the input fingerprint images are categorized as: (i) rolled full prints, by covering the nail-to-nail region of the finger (low resolution) [11,12]; (ii) plain prints, by covering flat region of the finger [11,13]; (iii) partial prints, captured from portable devices (high resolution) [12,14]; (iv) latent prints, acquired from touch surfaces (high resolution) [13,15,16,17,18,19,20]; (v) multispectral [21]; and (vi) contactless (2D and 3D) images [3]. Figure 1 shows variations between such conventional contact-based and contactless fingerprints.

The pipeline of a typical fingerprint biometric recognition system consists of four significant modules: the sensor module to acquire biometric images, the feature representation module, the matching module and the decision module where scores are computed for verification or identification of an individual [25,26]. There are certain challenges in each module of the fingerprint recognition pipeline, particularly sensors, image acquisition and feature representation. To select a biometric for an application, the aspects of accuracy, performance and security are of primary importance. Image quality is crucial to obtain desirable performance in the fingerprint systems where the image acquisition process plays a major role to capture quality images.

In the sensor module, the image quality of the biometric traits significantly affects the biometric system’s accuracy and performance. The image quality depends very strongly on the modality, such as whether it is a contact or contactless image. For instance, the presence of contamination on the finger or sensor surface and dryness or moisture on the finger severely affect the performance of the contact acquisition. Therefore, paying attention to imaging modality in the overall design is important to obtain better fingerprint systems. Further, the underlying principles of the operation of the sensor can lead to their own form of distortions during acquisition. Therefore, the fingerprint systems become challenging due to the inconsistencies and image variations and are vulnerable to external factors which create sensor interoperability issues [27].

On the other hand, feature extraction and decision modules solely depend on the quality of the image, image acquisition mode, photometric and geometric variations in contactless mode, standard pre-processing and enhancement techniques, invariant feature encoding or algorithms and matching algorithms or classifiers. Performance and accuracy of the systems are affected by the combination of the above factors. These factors should be analyzed in a comprehensive way to redirect and replace with alternatives in fingerprint biometric systems.

The existing reviews [28,29] of fingerprint systems only analyze the imaging techniques in fingerprint sensing existing for over a decade. However, the latest emerging trends, recent advances and their challenges in fingerprint sensing have been missed. Another recent article [30] restricted the review to deep-learning-based methods in contactless fingerprint recognition. Further, there is no work that explores challenges in fingerprint systems in the aspects of the sensor level and image-acquisition level with the whole range of fingerprints including plain, rolled, latent, partial and contactless 2D and 3D images under a common framework. Further, recent trends in the cross-matching of existing legacy fingerprint systems with contactless images has also been left behind, which calls for an investigation of the existing literature in the aspects of interoperability. Since the present reviews are either obsolete or restricted to a particular subset, this work aims to fill the gap identified in the present fingerprint systems by comprehensively analyzing the challenges in existing fingerprint systems ranging from contact to contactless in three major modules: (i) sensors; (ii) image acquisition; and (iii) interoperability and proposes alternate directions for the challenges encountered in contact and contactless imaging domains.

The remainder of the article is organized as follows: Section 2 analyzes various types of sensing technologies and their pros and cons, while Section 3 describes image acquisition, which covers different types of fingerprints captured from different sensing modes. Section 4 covers the cross-matching and interoperability issues of fingerprints. Alternatives for the challenges are outlined in Section 5, and the review is concluded in Section 6.

## 2. Sensors

**Figure 2:**
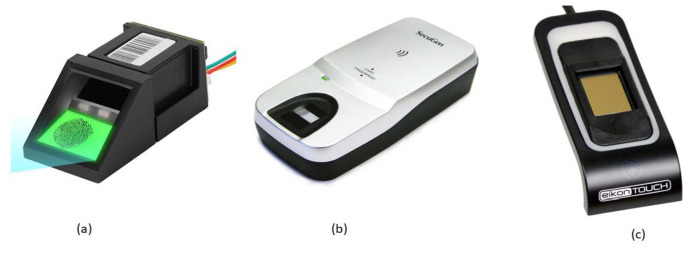
Variations of fingerprint sensors: (**a**) contact-optical [38]; (**b**) contactless-optical [39]; and (**c**) capacitive [40].

Fingerprint sensing is one of the most widely deployed techniques [31,32,33] in biometric sensing [34,35]. Fingerprint sensors can be categorized by the way the user interacts with them, such as contact, contactless, slap, partial, etc. [36]. Several sensing mechanisms have been used to detect the finger’s ridge-valley structure. Based on the underlying technology the sensors utilize, they are categorized into (i) optical sensors; (ii) capacitive sensors [37]; (iii) ultrasonic sensors; (iv) thermal sensors; and (v) pressure sensors. Figure 2 illustrates variations of contact optical, contactless optical and capacitive sensors.

Fingerprint sensing has been in development for decades. As it improves along with signal processing technologies, many applications are coming forward. The present competing technologies and related sensors have their own advantages and common shortcomings, such as electrostatic discharge (ESD), mechanical and thermal effects, direct exposure to the environment, discrimination between liveness and spoofing [29].

In fingerprint sensing, a lot of research has been aimed in two directions: (i) integration of fingerprint sensor with mobile phone; and (ii) advancement of stand-alone sensors. The mobile-phone-embedded sensors offer users more convenience by allowing sufficient space. Several prototypes have been introduced with different technologies towards mobile-phone sensor integration: (i) Qualcomm Technologies in the ultrasonic method [35,41,42,43]; (ii) Synaptics [44,45,46,47] in the optical method [48]; and (iii) on-display mutual capacitance in the capacitive method [49,50,51].

### 2.1. Optical Sensing

**Figure 3:**
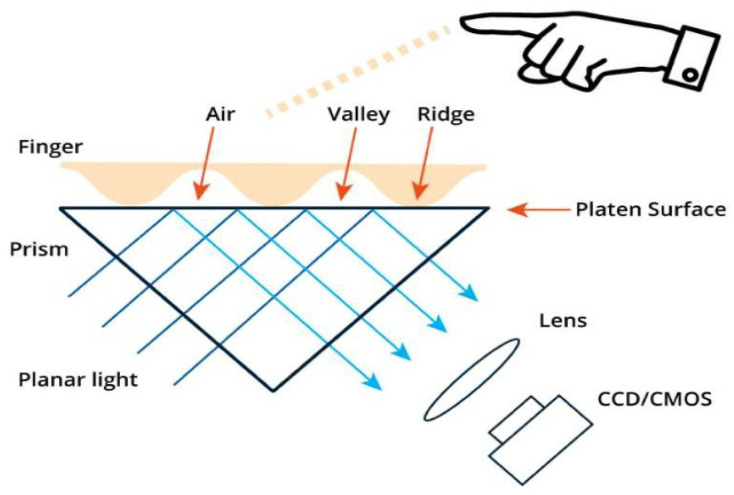
Optical fingerprint sensing architecture [53].

This is the oldest ’live-scan’ fingerprint sensor, in which a glass prism is illuminated when a finger touches the prism. Figure 3 shows the principle behind a typical optical sensor where ridges absorb the light, while the valleys allow the light to be reflected, and the reflected light is caught by CCD or CMOS sensors [52]. Optical sensors are low-cost fingerprint sensing devices comprised of the sub-techniques of reflection, transmission, sweep, TFT and electro-optical processes [28]. The primary technique the sensor uses is frustrated total internal reflection (FTIR) with an arrangement of a glass prism, a laser light and a CMOS or CCD sensor. It is not easy to arrange all of them in a portable form due to the very large focal length of small lenses. Further, it is not very easy to fool FTIR, and therefore, the scanners are vulnerable to imprecise fingerprint imaging.

Optical fingerprint sensing technology has been advancing over the years by enhancing the product features. However, the shortcomings related to the nature of FTIR can never be overcome. Even though optical sensing has advantages of producing low cost, good-quality images within a large sensing area and preventing electro-static discharge (ESD), it has the shortcomings that it cannot be shortened further due to the distance between prism and sensor, which limits the miniaturization. Further, it is sensitive to the contamination on fingers or platen, finger dryness and moisture, etc. Although it has shortcomings, several work based on optical sensing were demonstrated in the literature. The work in [54] demonstrated a 200 × 160-pixel CMOS fingerprint system-on-a-chip where column-parallel processors are embedded on it. Since the sensors are more sensitive to the dryness and moisture of the finger, the research group in [52] introduced a new fingerprint sensing approach for moisturized fingers by altering lens, prism design and optical-path structure.

On the other hand, present optical fingerprint sensing and development approaches do not work efficiently for latent prints which present on complex surfaces. Since latent prints exhibit extreme degradation, low image quality and image distortion, acquiring quality prints for reliable feature extraction from latent prints is challengeable. A recent work [55] proposed an alternate solution to overcome this issue. To acquire high sensitive and high-resolution latent images with less background interference, a dual-mode imaging setup with optical and electrochemical sensing is newly introduced. This method used conductive $Ti_{2}O_{3}$ black nanoparticles for latent fingerprint acquisition.

### 2.2. Capacitive Sensing

**Figure 4:**
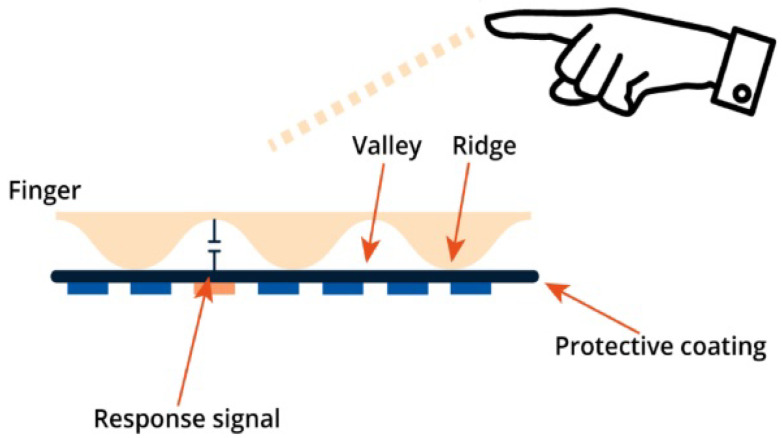
Capacitive fingerprint sensing architecture [53].

To overcome the drawbacks existing in optical sensing, capacitive silicon sensing has emerged. Capacitance is an electrical property present between two conductive surfaces [28]. In capacitive sensing, many capacitance plates are embedded in a chip. When a finger is placed, an electric charge is generated between the plate and finger. The sensor captures the capacitance varieties from the ridge-valley pattern. Figure 4 illustrates basic principle behind the capacitive sensor.

The existing capacitive sensors are classified as passive and active sensors [56] where the former conjoin finger and chip capacitance, while in the latter, the signal is directly placed on the finger to extract the fingerprint information.

The capacitive sensing technique is widely preferred in mobile and Internet of Things (IOT) applications due to the light weight, less power usage, reasonable cost-effectiveness, and convenience of embedding in the present applications [56,57,58]. There are several small and low-cost capacitive sensors identified in the literature [57,59,60,61,62,63,64]. However, most of the existing work experience the issues of finger sensitivity for wet and dry conditions and noisy environment. These factors significantly degrade the captured image quality. Different alternatives have been introduced in the past to enhance the captured image quality. The work in [63] used a local threshold level, while the research group in [59] exploited the voltage drop suppression.

A lot of research has explored integrating the fingerprint sensor in the mobile display and improving the stand-alone fingerprint sensing [57]. The research team [49,50,51] exploited on-display mutual capacitance to produce a prototype with finger-capture feature in mobile devices. However, this approach has a drawback related to the touchscreen, which requires a high voltage to overcome the panel and surrounding noise [50,51,65,66].

On the other hand, it is identified from the literature that many companies worked towards the stand-alone capacitive sensor for mobile and non-mobile based setups [67]. The stand-alone sensors play the key role in fingerprint recognition related alternatives. Samsung and other companies used stand-alone sensors due to less power consumption and cost effectiveness [68]. The work in [57] proposed a novel design for the stand-alone CMOS capacitive sensor using a new cell structure with effective features which enabled stable sensitivity for various finger conditions and noise.

The benefits of adopting capacitive sensing techniques are that it is smaller in size, consumes less power and is user friendly. However, it is vulnerable to strong external electrical fields, ESD and is expensive. These shortcomings limit the usage of this technique and look for alternate means [28] for replacement in fingerprint sensing.

### 2.3. UltraSonic Sensing

**Figure 5:**
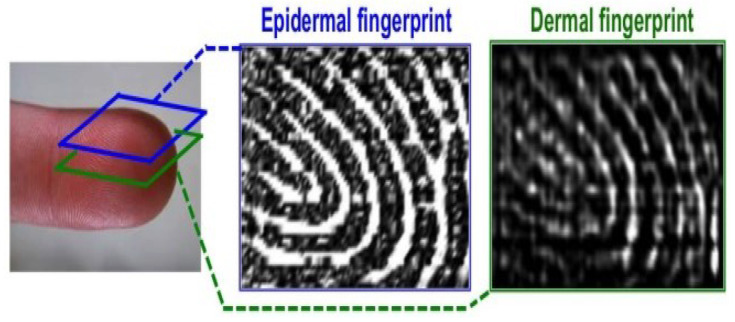
Ultrasonic finger images acquired from epidermal and dermal layers. Reprinted with permission from Ref. [33] 2016, IEEE.

Ultrasonic fingerprint sensing can be an alternate means for the existing fingerprint sensing modes. The principle behind the ultrasonic sensing is a medical ultrasonography where high frequency sound waves penetrate into the skin’s epidermal layer. Since dermal and epidermal layers have similar features, as illustrated in Figure 5, the reflected measures are used to capture the finger image using piezoelectric materials [28].

**Figure 6:**
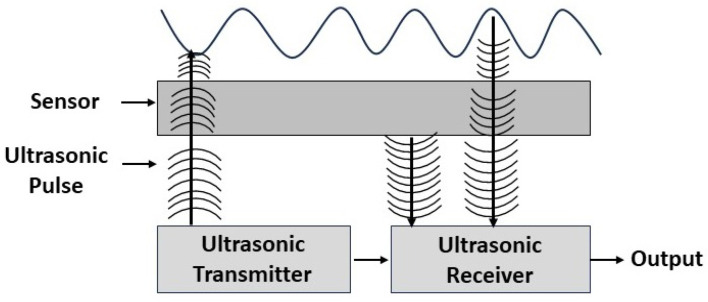
Ultrasonic fingerprint sensing principle [72].

The sensor has sender and receiver modules where the former sends acoustic signal towards the finger, while the latter acquires the results when these signals backlash the fingerprint surface. The principle behind the ultrasonic sensing is illustrated in Figure 6. This sensing technique has several advantages, such as consuming low power, being insensitive to contaminants and light and being easily transmitted through metal and glass [69,70]. Therefore, it has been attractive for use in smart phones, IOT and augmented and virtual reality devices. To introduce ultrasonic methods in cell phones, Qualcomm technologies are exploited [35,41,42,43], where the sensor is placed on screen using 700–800 µm penetration capacity. Due to the higher cost of Qualcomm sensors, it is presently available on the latest top-branded mobiles only [71].

This review comprehensively explores the advances devoted in ultrasonic sensor technologies emerging from the classical piezoelectric transducers to the most recent CMUTs and PMUTs, that are completely missed in the existing sensor-based reviews. Piezoelectricity is the fundamental of ultrasonic sensing, and later on, lead zirconate titanate (PZT), which is strong and has piezoelectric properties, was discovered. The overall ultrasonic sensing technique is divided into two categories: (i) ultrasound imaging techniques where pulse-echo imaging and impediography methods are approached and (ii) transducer technologies where piezocomposite transducers, capacitive micromachined ultrasonic transducers (CMUT) and piezoelectric micromachined ultrasonic transducers (PMUT) are explored. The CMUT comprises of a high number of cells with vibration [73], whereas the PMUT devices composed of two key piezoelectric substances: aluminum nitride (AlN) and PZT [34].

**Figure 7:**
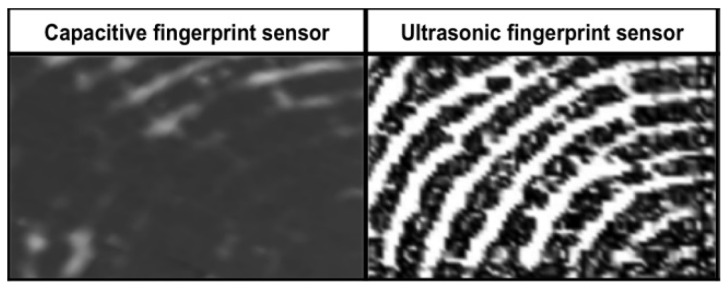
Fingerprint capture from capacitive vs. ultrasonic sensors. Reprinted with permission from Ref. [33] 2016, IEEE.

Using the pulse-echo imaging technique, the first experiment was for live-scan fingerprint capture [74], where external focusing lenses were used and the transducer produces a 0.2 mm spot size. In a later work, a transducer with 50 MHz was used, which permits a resolution of 1000 dpi [75], enabled the showing of sweat pores that are not be visible from optical sensing. Figure 7 shows the difference between capacitive and ultrasonic sensing. However, the work in [75] has a limitation of long acquisition time, and the issue was addressed by integrating cylindrical scanning in a later work [76]. The same research team then demonstrated the capture of fingerprint patterns from under-skin layers without any skin deterioration as well [77].

In early days, bulk piezoceramic transducers were exploited in classical ultrasonic fingerprint sensing with XY mechanical scanning in mobile devices. However, it failed to reach the portable device constraints such as the size and cost. Therefore, the research explored CMUT- and PMUT-based approaches [78]. The first CMUT-based fingerprint sensor was presented in 2010 [73], where remote electronics were used to read 192-element 1D line-scan array while the 2D image was acquired using mechanical scanning. However, the interface with complexity between the sensor array and the electronics again failed to reach the size constraint of the portable device. Even though the CMUT approach has the limitation, the research team [79,80] investigated the possibility of capturing fingerprint images using the impediography method with CMUT sensing.

The advantage of using ultrasonic sensing is that it has higher reliability compared to other sensing schemes as it computes the difference between the acoustic impedance of the finger ridge-valley structure. However, a shortcoming of higher cost was experienced in the past. To overcome the cost issue, the research team in [81] demonstrated a setup by introducing the PMUTs, which were built using micro-fabrication, resulting in a low-cost system, which raises privacy and security in consumer electronics. In a later work, short-range imaging was experimented with using PMUTs arrays [82]. However, with the absence of electronics integration, the individual display of the PMUTs in an array is discouraging. To achieve portable device constraints such as size and cost, there are other techniques needing to be integrated with the existing, and the research team in [83] presented the initial demonstration of a pulse-echo ultrasonic sensor by integrating MEMS and CMOS wafers to meet the portable size constraint and other features such as rich signal, less power and a less voltage interface. The similar technique was experimented in a later work as well [32]. They implemented the ultra sensing by linking MEMS and CMOS wafers to reach the compact size in addition to the features of rich signal efficiency, low tensile strength and a low-voltage interface.

Apart from the above demonstrations, 3D fingerprint images were acquired using commercial systems such as Technos-Esaote, with water as a coupling medium. The research team in [69,83] introduced a sensing approach using a AlN PMUT 2D array with a 24 × 8 elements integrated with portable device electronics and CMOS technology, resulting in a total area of 2.3 × 0.7 mm${}^{2}$. The work in [84] experimented with a 65 × 42 element sensor using PMUTs integrated with CMOS. The array size was raised up to 110 × 56 elements, which resulted in a fingerprint size of 4.73 × 3.25 mm${}^{2}$ as an upgraded version of the sensor [33,71,84,85,86,87]. This helps to capture fingerprints at two layers (epidermis and dermis), as shown in Figure 5.

The research team in [88] performed a feasibility check using 1–3 piezocomposite ultrasonic transducers to detect fingerprint patterns through pulse-echo methods. Apart from piezoelectricity, PZT brought an effective impact in ultrasonic applications. Since the presence of the piezoelectric property in the PZT, PMUT has been developed based on the PZT, where the transducer comprises of an array of 50 × 50 PMUTs with the fabrication of a sol–gel PZT technique. In a later research, the team [31] experimented with a large-area (20 × 30 mm${}^{2}$) sensor with diverse functionality by integrating a thin sensor and thick (>1 mm) mobile display. This helps to acquire fingerprint features and the finger touch pressure level effectively.

The significance of the ultrasound sensing over other technologies is that it has the capability to acquire a large volume of the finger, supports wet fingers and results in several benefits: (i) distinctive feature extraction; (ii) flexibility of finger touch location due to the large area; (iii) convenience of use; (iv) lack of vulnerability from surface contamination and humidity [34]. Further, pulse-echo ultrasonic imaging measures images at multiple depths from the sensor and beneath the epidermis, which resulted in the image’s resistance to spoof attacks.

### 2.4. Pressure Sensing

**Figure 8:**
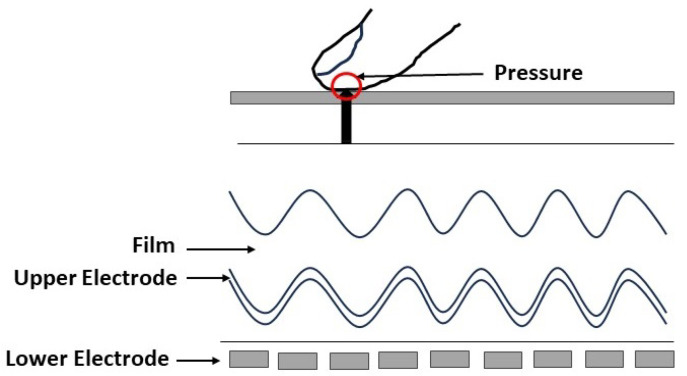
Principle behind the pressure sensor [90].

The piezoelectric effect is the principle behind the pressure sensor, where a small amount of current is generated when there is a physical touch of a finger with the sensor surface which made up of a non-conducting dielectric material. The effectiveness of the current relies on the finger pressure, and it varies as only ridges contact the sensor. Figure 8 shows the principle behind the pressure sensor. The size and resolution of the pressure sensors are similar to the capacitive sensor. However, the material used in this technique has low sensitivity to acquire fingerprints accurately. Further, it is less sensitive to wet and dry conditions of the fingers.

### 2.5. Temperature Differential Sensing

This sensor is operated based on the temperature difference which can be generated when two surfaces are in contact [28,89]. It is composed of pyro-electric material that generates current with the conversion of temperature changes into a voltage. ’Atmel FingerChip’ is one of the most common thermal sensors, exploited in many publicly available fingerprint data acquisition sensors such as FVC2004 (DB3) and FVC2006 (DB3) [89].

### 2.6. Optical Coherence Tomography (OCT) Sensing

OCT is the latest, optical, non-destructive, non-invasive high-resolution and significant method for biomedical investigation [91]. With the recent advancement, biometric applications have used this technique in 3D fingerprint image acquisition [92,93,94,95].

The fingerprint consists of epidermal and dermal layers to represent the surface and internal regions of the fingerprint. Internal fingerprint surfaces are not sensitive to wet and worn conditions and have anti-counterfeiting capacity compared with 2D finger images. Even so, it is challenging to extract features from both layers due to the noise and low contrast in contactless domains. To overcome this nature, the research group in [94] introduced a spectral domain-based OCT setup which helps to extract surface and internal features of the fingerprints effectively.

It is obvious that the OCT opens up a new trend in image acquisition for fingerprint recognition as it has the capability to acquire in-depth information of the finger. The research team in [93] investigated different types of fingerprints such as normal, worn-out, artificial and degraded using a customized OCT device. Reconstruction of three subsurface layers is proposed using the skin layer information. Later, pixel-based fusion from the three subsurface layers was performed, which resulted a robust fingerprint recognition.

The principle behind the OCT is interferometry, where light reflected from both finger and reference mirror is merged using a detector. Spectral modulation helps to acquire depth information. The advantages of using OCT is that it extracts information from the finger up to 2 to 3 mm. Since it is invariant to skin damage and helps to reconstruct the finger, it is less vulnerable to spoof attacks.

### 2.7. Radio Frequency Sensing

This technique can be used to detect finger live layers. When capacitive sensors fail to capture fingerprints, they can be replaced with RF technology. The issue in this technique is that a gummy finger still can imitate a real finger and fool the sensor [96].

## 3. Image Acquisition

Finger-sensor surface contact is a key concept in fingerprint image acquisition. Physical contact is the most adopted image capture approach which is currently in use in many applications. Contact sensors such as optical with CCD, capacitive, and digital scanners have been used for contact-based image acquisition. Optical sensors are beneficial for fingerprint recognition with high-resolution images, However, they have large physical volumes with light sources, detectors and optical parts. The work in [97] demonstrated an ultrathin contact-based compact camera as an alternative, where the CMOS sensor is integrated with a microlens array and multiple block layers.

There are different scenarios to acquire contact-based fingerprints using various sensors. While the imaging techniques are advancing with sensor variations, the output of the fingerprint sensors are classified as (i) rolled full prints covering nail-to-nail area [11,12]; (ii) plain fingerprints covering flat regions [11,13]; (iii) live-scan swipe or partial fingerprints captured from portable devices [12,14]; and (iv) latent prints captured from crime scene surfaces [13,15,16,17,18,19,20]. Each acquisition mode can have different physical finger placement with the sensor surface and therefore exhibits various challenges which call for alternatives.

### 3.1. Rolled and Plain Fingerprints

Rolled and plain fingerprints are a major contribution in contact-based image acquisition [11,98]. The former is acquired while pressing the finger and the whole surface of the fingertip is rolled over to scan, whereas the the latter is captured without rolling the fingertip [99]. The low-resolution rolled and plain fingerprints are obtained from a touch-based scanner where the finger pad is flattened against the image acquisition surface for plain images [100]. There are several forms of distortion during contact capture as the ridges are unambiguously recorded by this acquisition.

**Figure 9:**
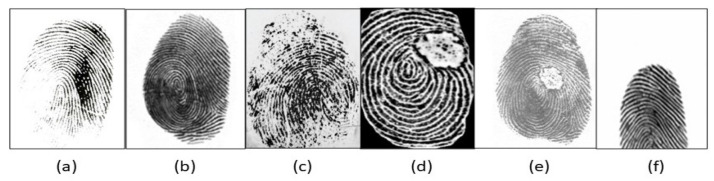
Typical challenges in contact-based fingerprints: (**a**) blurry image [102]; (**b**) distorted print [103]; (**c**) degraded print [104]; (**d**,**e**) deformed prints [104]; (**f**) partial finger capture.

In general, these touch-based prints have the shortcomings of noise in images and the skin’s wet and dry conditions [99]. There may be a contamination impurity on the surface as well. Because of the variations on finger pressure with sensor, non-linear distortion also can exist. Further, the factors of lack of resolution, lack of ridge detail and poor contrast regions, less inter-class (fewer differences between two different fingers) and large intra-class variations (multiple impressions from a single finger) as shown in Figure 9, yield a poor performance in contact-based fingerprint systems [101].

On the other hand, for the rolled fingerprints, 3D finger structure is converted into a 2D plane by rolling the finger across the capture surface [100]. Rolled fingerprints have comparatively a larger fingerprint area than flat fingerprints, which helps to extract more minutiae. They are demanded in many fields, including military environments and civil applications. The challenge that has been experienced for a long time is the distortion due to too much or insufficient pressure of the finger with the capture surface or ink during finger registration, which needs human supervision. Due to the elastic deformation of fingertips, the mosaicking gaps can be visible and locating them is difficult [11].

For the inked fingerprint capture, over- and under-inking and sliding of the finger with the surface also can be possible. The digital image of the print on the card is a representation of the finger captured from inked impressions, which is a second-order representation of the ridge structure of the finger surface [100]. Therefore, the degree of fingerprint quality is constrained by the defects that occur before the finger image digitization process. Additional errors also can be possible from optical scanning during the inked impression/digital form image conversion.

### 3.2. Latent Fingerprints

Latent prints are used more in law enforcement and forensic applications. Most techniques for acquiring these prints use a method by contaminating the fingerprint with chemicals. This makes the latent unusable for further evaluation. Alternatively, they are acquired from high-resolution cameras to enhance the visibility of information from the touched surfaces where the latent print presents [16]. Though there are different types of fingerprints used, latent prints become dominant and broadly exploited as evidence in law enforcement, mostly used in Federal Bureau of Investigation (FBI) databases. However, most forensic finger-mark evidence has not been scientifically validated yet [105]. The comparison of finger-marks with the reference fingerprints is becoming challenge in latent fingerprint recognition, and therefore, automated fingerprint identification systems are still in the growing stage, which needs an enhancement to be compatible with the environment of latent vs. digital image matching.

The challenges in latent prints are computational: (i) lack and poor quality of ridge information in partial latents; (ii) background noise; (iii) lack of contrast and blurring and poor clarity due to the distortion; (iv) having fewer minutiae due to small capture area of a finger; and (v) overlapped fingerprints [20]. The presence of fewer minutiae, having poor clarity of ridges and skin distortion make the latent fingerprint systems practically slower, which demands further investigation of the latent fingerprint systems for automated methods. On the other hand, the marks obtained from crime scenes can vary and range from partial finger, palm or entire hand. Sometimes, forensic experts cannot identify the region where the print actually locates on a hand of an individual. Further, cross-modality matching of fingerprints transferred from the rigid objects with direct finger photos also offers challenges.

### 3.3. Partial Prints

Advancement of high-resolution sensors attracted fingerprint biometric recognition in recent years. Civil applications adopt plain fingerprints using consumer electronic devices. Because of the trend of using miniaturized portable and lower-cost fingerprint scanners, people tend to move towards partial prints. Classical full-size and low-resolution fingerprints differ from high-resolution partial prints which result in smaller areas of the prints. With the use of larger print areas in rolled and plain fingerprints compared to partial fingerprints with smaller areas, partial fingerprint matching has received attention in improving performance these days [14,106]. However, there are three significant challenges identified in the use of partial fingerprint systems.

**Figure 10:**
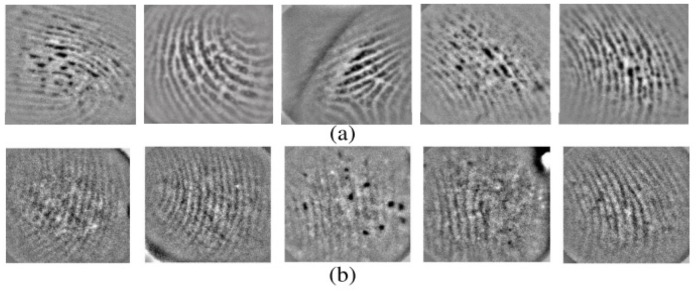
Partial fingerprints acquired from different fingerprint skin conditions using optical sensor (800 ppi): (**a**) images acquired under normal humidity; (**b**) images acquired under dry skin. Reprinted with permission from Ref. [99] 2022, IEEE.

**Figure 11:**
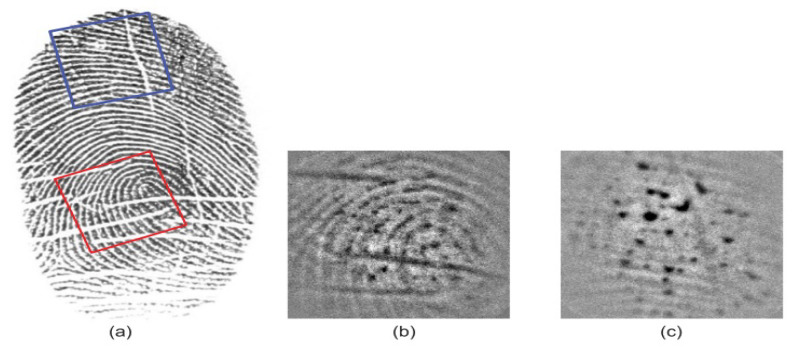
Full fingerprint vs. corresponding partial fingerprints: (**a**) full fingerprint acquired using capacitive sensing with 500 ppi resolution; (**b**,**c**) corresponding partial prints with respect to red and blue squares on the full fingerprint. They are acquired using under-screen optical sensing with 800 ppi resolution. Reprinted with permission from Ref. [99] 2022, IEEE.

1. Small area limits the feature points
  The size of the scanners used for partial prints is only 12.7 × 16.0 mm${}^{2}$ [107]. The study proves that decreasing the active area limits the feature points acquired from fingerprint capture region and decreases the performance gradually [108]. Therefore, the fingerprint recognition methods significantly degrade, which urges the use of intelligent portable devices, especially for wearable devices.
2. Fingerprint image quality
  Since the quality is an important concept in fingerprint recognition, low-quality images are even worse in partial prints, as illustrated in Figure 10 and Figure 11. The practical issue is that when we focus on portability, then image quality is compromised, and therefore, there is a trade-off issue between these two qualities in partial fingerprints. The study in [109] shows that partial prints can have 3–15 minutiae within a small area, which is comparatively lower than in a large area. Because of the presence of or lack of minutiae or feature points due to the small print size and poor quality, specialized algorithms are required to extract pores and ridge contour features.
3. Image capture condition
  There are several varying conditions such as humidity on skin and changes in lighting and temperature during image capture. Therefore, adopting a generalized algorithm for these varying conditions also can be a challenge, which urges the biometric industry to find alternative solutions.
4. Geometric variations
  Geometric variations and need for image pair alignment during the matching process are other factors which cause a negative impact on the partial-fingerprint-based recognition systems [110].

In a summary, direct physical finger-sensor surface contact yields a set of challenges. Non-linear deformation arises due to the nature of the elasticity of the skin and significantly degrades the matching performance. The other concern with the contact-based approach is that the latent of the previous attempt left on the surface can be copied and taken for illegitimate use. This leads to a security risk through spoof attack. Further, the challenges such as contamination with the surface, hygienic issues, image distortion and elastic deformation due to the pressure [22], surface artifact or wetness and a lengthy time consumption for scanning also result in the call for an alternate approach for contact-based acquisition. Therefore, it is necessary to move towards the contactless domain, which uses several advanced strategies to eliminate these issues by using digital cameras to capture adequate resolution and quality prints. Table 1 summarizes fingerprint image acquisition modes and their related issues.

**Figure 12:**
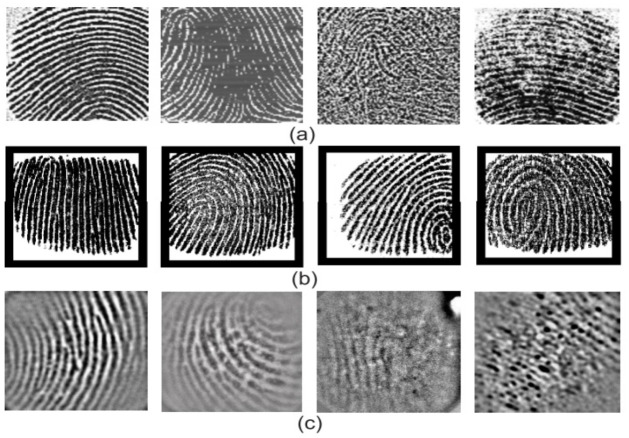
Quality variations of partial fingerprints acquired from different sensors; (**a**) FVC2006 DB1; (**b**) AES3400; (**c**) ZJUPartial. Reprinted with permission from Ref. [99] 2022, IEEE.

Further, contact fingerprints can have different challenges due to the variations in image acquisition modes. Sensor variation generates different output images: full, live-scan partial prints and latent. This challenges the feature representation and matching algorithms by demanding invariant features for geometrical variations. In addition, there are imaging-level variations in partial fingerprint recognition. Fingerprint sensor interoperability is the main concern due to the variations in image acquisition because, in practice, the automated fingerprint identification process needs to compare and match the fingerprints captured from different devices. Figure 12 illustrates quality variations in partial prints acquired from different sensors.

In the image acquisition module, output image quality from the contact-based sensor significantly affects the fingerprint system’s accuracy and performance. The performance of the fingerprint system is evaluated based on false accept rate (FAR), false reject rate (FRR) and equal error rate (EER) during matching of two prints [111]. The image quality challenge can be mitigated by paying attention during the image acquisition process. Some of the precautions in quality and safeguard health can help to eradicate them. For instance, wiping or cleaning the contact surface before image capture and cleaning the finger tip before and after use can be some steps. Further, using image quality estimation algorithms can help to select quality prints and discard the useless images before entering into the system.

Pre-processing using standard enhancement techniques can also help in this regard. Structured noise generally exists on fingerprint due to stains, lines, overlapping background prints. These environmental effects in degraded fingerprints can be overcome by its restoration using image-processing-based or learning-based enhancement models. The generative adversial network (GAN) is the state-of-the-art image generation or enhancement techniques. The researchers in [112] recently demonstrated that the channel refinement-generative adversial network, which is one of the degraded fingerprint restoration methods, outperformed than the GAN and classical image processing enhancement.

Invariant feature representation and matching also can be investigated to preserve the performance of the system. However, in practice, matching process needs to be performed between different types of image pairs: (i) full fingerprint vs. partial captured from different sensors [7]; (ii) latent vs. full print; (iii) latent vs. contactless image; and (iv) contact vs. contactless [113]. Therefore, the algorithm implemented for a fingerprint system that uses images acquired from a particular mode of acquisition cannot be successful in other mode of prints. This calls for an investigation of the existing fingerprint systems to inter-operate feature encoding and matching algorithms.

### 3.4. 2D Contactless Fingers

Contactless fingerprint recognition has received an alternate means to contact-based systems and extra attention due to the hygienic nature of the sensor. The pandemic also encourages the actual necessity of the contactless biometric systems. However, investigations in contactless fingerprint domains were initiated with the 2D approach early in the past and have been continuing to date in 3D-sensing technology [114]. Recent advances in smartphone cameras also motivated the capture of contactless finger images due to less cost and the portable unconstrained nature of the devices [115,116,117].

Contactless fingerprint images are an optical representation of 3D structure of the finger surface onto a 2D plane. Contact fingerprints yield a first-order representation of friction ridge surface by absorbing the light and dark of the image [100]. However, in contactless setupss, the light and dark of the finger friction on the surface, being modeled by illumination since reflection and shadow, lose the coupling between finger image and the capture device. Therefore, contactless acquisition of fingerprints deviates from contact-based image acquisition technologies. The actual finger friction ridge surface is in 3D topography, and contactless finger images are in 2D representation of 3D structures.

**Figure 13:**
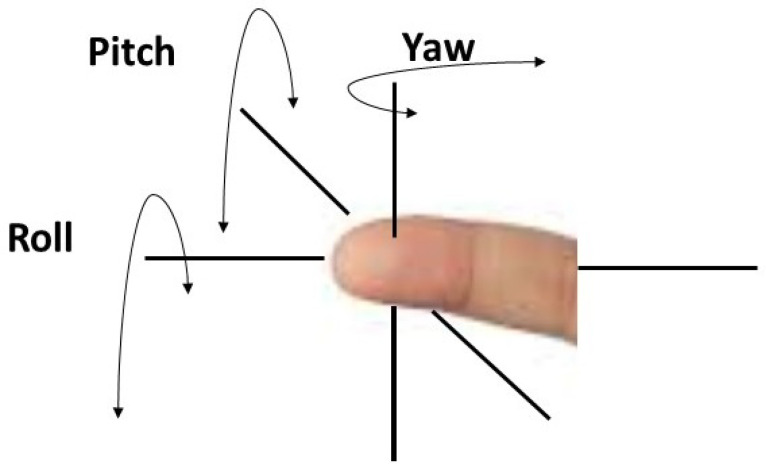
Possible distortions arise from contactless nature.

In 2D contactless image acquisition, one or more fingers are presented on a sensor. The sensors can range from (i) prototype hardware for research purposes and (ii) general purpose devices with customization of the image capture requirement [25]. In the prototypic hardware design approach, box-like setups with LEDs were used in the early days to maintain uniform illumination without environmental factors. Some of the setups used finger guidance or fixed-finger placement. The common adjustments made in these setups were strong illumination and small distance between the sensor and the finger. However, the distortions experienced in all these constrained setups are illustrated in Figure 13.

For the general purpose devices, web cams, smart phones and digital cameras were some of the commonly used devices in the past. Due to the low cost and user convenience, researchers used web cameras which adopted manual capture without additional illumination since external illumination severely impacts the system performance [118]. Use of smart phones is one of the widely adopted techniques for single-finger image capture [119] as they are widely available with quality cameras and show quick response. Since the device has additional features such as auto focus, macro lens, flash lights and on-screen finger guidance, a convenient automatic capture of finger images is enabled using smart phones.

Digital cameras are another means to acquire contactless 2D finger images. Image sensors based on white and LED color are primarily used in these setup [25]. Since there are some advantages of using multi-finger over single-finger biometric systems, these devices are widely used to capture multiple fingers as they efficiently help to extract features from all five fingers [2,120,121]. Much contactless finger image capture work in the literature is demonstrated under various environmental factors [115,122,123,124,125], for instance, different background, range of lighting, indoor and outdoor image capture, etc. Table 2 illustrates an overview of the existing contactless 2D-imaging modes and the features used with constraints.

Overall, touchless 2D systems, lighting sources and imaging cameras are placed on the same side, and the image is captured based on the illumination reflected on the finger ridges. In some cases, illumination sources are placed behind the fingernail side where the illumination penetrates the fingerprint and results in the final image. Even though the contactless systems advance over many classical features of contact-based systems, they suffer from low contrast between ridges and valleys, which incurs well-established enhancement techniques for feature extraction.

Further, alternative means for image acquisition are required in place of 2D devices. An advanced setup with prototypic hardware is demonstrated in the recent past with CNN feature extraction [126]. The prototypic hardware captures finger image usinga Raspberry Pi NoIR (no infra-red) camera. Further, multispectral [21], multiview [22,127] and 3D touchless technologies have been investigated with the recent advancement strategies.

**Table 2:** Summary of existing contactless 2D-imaging modes used for image capturing.

Reference	Image Capture Mode	Features and Constraints
Genovese et al. [128]	Digital camera, green LED illumination	Level-3 features
Sankaran et al. [115]	Smart phone	Unconstrained images, manual image capture
Canrey et al. [121]	Smartphone	Slap hand, on-screen guidance for multi-finger image capture.
Deb et al. [129]	Smart phone	Two fingers such as index and thump fingers, two commercial apps on 3 smartphones
Birajadar et al. [130]	Smart phone	On-screen guidance
Kumar and Zhou [131]	Webcam	Low cost, no spatial illumination
Ravi et al. [118]	Webcam	Semi-mobile, fixed, auto-focus, noisy background
Weissenfeld et al. [132]	Prototypical hardware	Multi-finger capture
Kauba et al. [133]	Smart phone	Contact-contactless comparison
Jannis Priesnitz et al. [25]	Smart phone	Multi-finger capture
Attrish et al. [126]	Prototypic hardware-RaspberryPi No infra red	Single-finger photoMinutiae and CNN features
Akmal-Jahan et al. [2,120,122]	High-resolution Digital Camera	Two fingers: index and middle finger imagesMultiple finger segmentsRidge orientation pattern

To mitigate the issue of performance drops in contactless matches, the image should be carefully treated in each module of the fingerprint system pipeline. For instance, in pre-processing, standard quality estimation mechanism and state-of-the-art image enhancement techniques can be practiced, while in feature extraction, deep and invariant feature representation can be employed [126]. For instance, in image enhancement and restoration, generative adversial network, which is the state-of-the-art method for image generative problems [112], can be employed. For invariant feature representation, ridge orientation pattern can be used as it will not be affected by sensor differences [7]. Deep-learning-based features provide a promising result compared to other hand-crafted features in recent fingerprint experiments [22,134,135]. Further, contactless high-resolution images can also be employed for rich ridge features which can show clear details of the texture [2,120,122].

### 3.5. 3D Contactless Fingers

For the 3D fingerprint capture, researchers have exploited some prototypes experimented with in laboratories. They comprise different strategies and techniques: (i) structured light scanning; (ii) photometric stereo techniques; (iii) stereo vision; (iv) ultrasonic sensing [136]; and (v) optical coherence tomography (OCT). Table 3 depicts a summary of recent contactless 3D-imaging strategies.

**Figure 14:**
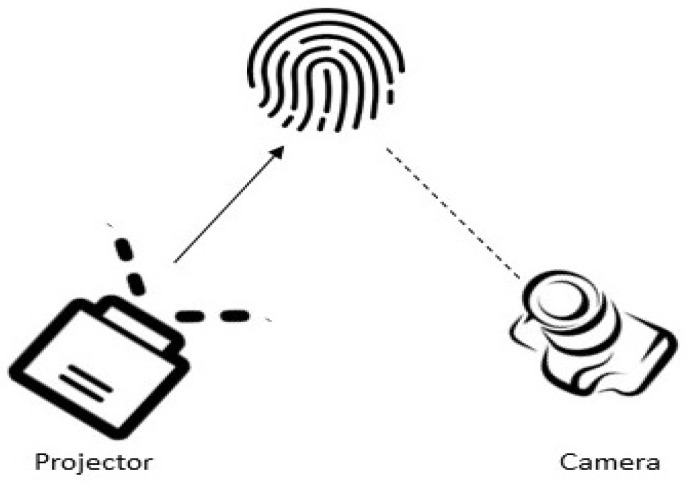
3D fingerprint capture using structured light scanning.

**Figure 15:**
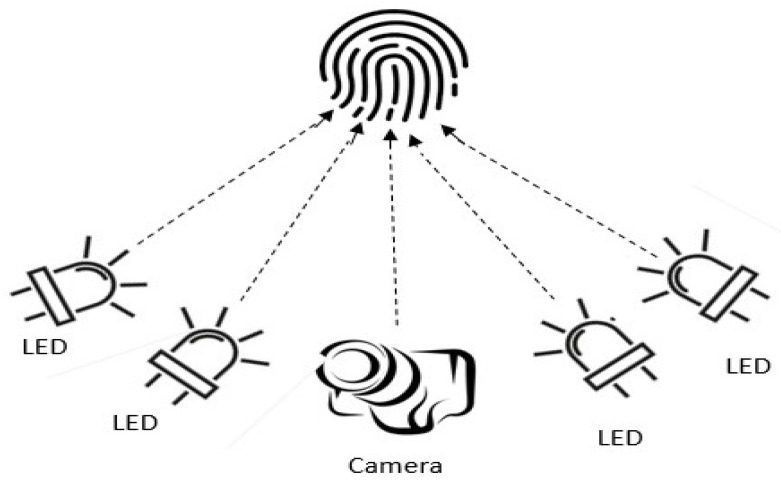
3D fingerprint capture using photometric stereo techniques.

**Figure 16:**
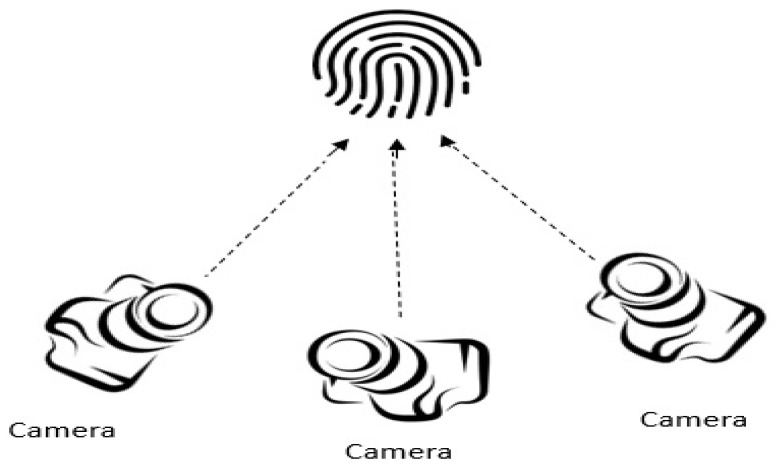
Acquisition of 3D finger image using stereo vision.

1. Structured light scanning
  In this approach, a set up with multiple cameras and a projector is arranged to capture 3D images. It is noted that multiple 2D images are acquired based on pattern illumination where 3D depth information is computed based on the triangulation using the point correspondences between images [137]. Even though this approach helps to obtain detailed and accurate ridge-valley and 3D depth information, it requires a complex and expensive hardware setup [138]. Figure 14 illustrates the basic setup of the structured light scanning.
2. Photometric stereo techniques
  In this approach, many 2D images are acquired under the condition of various illumination from a constant viewpoint using a high-speed camera. The main principle behind this technique is that time of flight (ToF) and surface reflectance between fingerprint and light source are computed [139]. A setup with a camera and multiple LEDs is used in this approach, as illustrated in Figure 15. It is noted in the literature that photometric stereo is the widely adopted technique among all other 3D contactless approaches. The main advantage is the cost effectiveness of the setup. Further, it results in high-quality ridge details of the fingerprints [136,140,141]. Fingerprints are reconstructed when fixed illumination is given using 3D surface orientations. However, unconstrained finger movements are experienced in this strategy, which decreases the reconstruction precision of the fingerprint system.
3. Stereo vision
  In this approach, two or more cameras are used from different views to capture images [136,142,143]. 3D depth information is computed using the corresponding points based on the triangulation. This information is used to reconstruct the 3D images. This process has some advantages such as simplicity, affordability, and compact setup. However, existing approaches in the literature using this strategy have a drawback of long time consumption due to the additional computation of the correspondences between pixel points [10]. Figure 16 illustrates the basic setup of stereo vision scanning.
4. Ultrasonic sensing
  Ultrasonic imaging is one of the 3D contactless imaging techniques where acoustic pulse moves forward (transmitter to fingerprint) and backward (to receiver) directions [69,86]. There are several research using the ultrasonic finger image capture in the literature [31,32,33,69,70,76,86]. Acquiring high-resolution images is the significant advantage in ultrasonic sensing. However, large-volume hardware structures lessen the attraction compared to other 3D contactless strategies. It is noted that ultrasonic sensing-based 3D contactless imaging needs further investigations and directions in future.
5. Optical Coherence Tomography (OCT)
  OCT is the latest, optical, non-destructive, non-invasive high-resolution method for 3D fingerprint image acquisition [92,93,94,95]. The fingerprint consists of epidermal and dermal layers to represent surface and internal regions of the fingerprint. Internal fingerprint surfaces are not sensitive to wet and worn conditions, and have anti-counterfeiting capacity compared with 2D finger images. Even so, it is challenging to extract features from both layers due to the noise and low contrast in contactless domains. To overcome this nature, the research group in [94] introduced a spectral domain-based OCT setup which helps to extract surface and internal features of the fingerprints effectively.
  It is obvious that the OCT opens up a new trend in image acquisition for fingerprint recognition as it has the capability to acquire in-depth information about the finger [93]. The principle behind the OCT is interferometry, where light reflected from both finger and reference mirror is merged using a detector. Spectral modulation helps to acquire depth information. The advantages of using OCT is that it extracts information from the finger up to 2 to 3 mm. Since it is invariant to skin damage and helps to reconstruct the finger, it is less vulnerable to spoof attacks as well.

**Table 3:** Summary of recent contactless 3D-imaging strategies.

Authors and Year	3D Imaging Strategy
2018 [140] 2013 [141]	Photometric stereo
2020 [144] 2019 [145] 2017 [146]	Structured light imaging
2021 [3] 2015 [142] 2014 [147]	Active and passive stereo camera
2020 [31] 2020 [32] 2017 [86] 2016 [33] 2015 [69] 2015 [70]	Ultrasonic sensing
2022 [95] 2019 [92] 2020 [93] 2020 [94]	Optical coherence tomography (OCT)

Even though the contactless nature of imaging has advantages over contact prints, they have their own challenges. The majority of them are photometric variations such as lack of ridge-valley contrast, irregular illumination, and geometric variations, distortion due to scale change and varying rotational changes such as roll, pitch and yaw of the finger, as illustrated in Figure 13, and different backgrounds with noisy environments as well. Therefore, the fingerprints acquired from the contactless nature need to overcome the challenges to compete with the similar level of accuracy in fingerprints that are acquired from contact-based methods. Apart from the geometric and photometric nature of the capturing images, fingerprint interoperability is also a major concern where cross compatibility of digital images is matched with their counterparts of the contact prints.

It is noted that there are currently no accepted industry standards for 3D representations of fingerprints that demonstrate compatibility with legacy fingerprint databases [100]. Further, contactless image acquisition adopts image processing techniques to convert RGB images into greyscale and binary form, which results in a third-order representation of the image. Therefore, handling contactless images is comparatively complex with the counterpart of their contact prints. Therefore, there should be an investigation to evaluate the interoperability of the contactless representations with legacy fingerprint impressions.

## 4. Cross-Matching and Interoperability

Cross-matching is a process of acquiring an image from one mode of capture and matching it with an image acquired from another mode. This has the advantage that the fingerprint systems can abstain from the process of re-enrollment of already registered users [25]. There is a recent advancement of matching images captured from contact-based and contactless in a single system [148,149,150,151]. Cross-matching is a key concept when any governmental large-scale projects that work with their citizens’ existing contact-based information by extending them in contactless domains [22]. However, storing legacy database with contact-based fingerprints and adopting contactless system from the existing system is a key challenge [150].

Advancement in fingerprint technologies and increase in fingerprint applications made a way to use different fingerprints captured from various sensors. This creates an issue in fingerprint system’s performance. For instance, applications such as security agencies, service providers and forensic departments might use fingerprints captured using a particular sensor. Later, authentication and verification can be performed by different types of sensors, which leads to a problem [152]. The significant factor which limits the use of contactless fingerprint technology is intercompatibility with their counter touch-based fingerprints.

Since there are more centralized fingerprint identification systems deployed these days [152], there is a need to acquire input sources from various fingerprint sensors, which requires more attention of the emerging problem of interoperability. The interoperability problem can be analyzed in two different directions: (i) cross-matching of fingerprints acquired using contact-based sensors with different techniques [7,152]; (ii) cross-matching of finger images acquired using contact-based and contactless devices [22,113].

In the recent past, several studies have explored the matching of contact-based slap images with contactless images to enhance the compatibility of matching [22,36,113,129,153,154,155]. A few approaches focused on cross-matching issue are identified: (i) fusion of existing fingerprint-matching methods [27]; (ii) non-linear distortion [113]; (iii) co-occurrence of ridge orientation (Co-Ror) [7]. Experimental methods in [7,152] satisfied the interoperability between contact-based fingerprints captured using different techniques. However, challenges exist in contact–contactless fingerprint matching, and none of them achieved the accuracy similar to the accuracy of contact–contact matching.

It is clear that the contact-based prints should compromise the issues of deformation due to non-uniform pressure, latent prints and noise present on the surface, while contactless should compromise the issues of geometrical and photometrical variations and deformation due to the movement. Differences in image formation and image distortion are the two major factors impacting the performance drop during the cross-fingerprint matching [150]. In the image formation, ridge and valley contrast is a significant cause. Sensors record ridge-valley reflections towards the light in contactless fingerprint capture, whereas high-contrast between ridges and valleys results in contact-based capture. For the distortion issue, pose variations and elastic distortions can be experienced for the same finger in contactless and contact-based capture, respectively.

**Figure 17:**
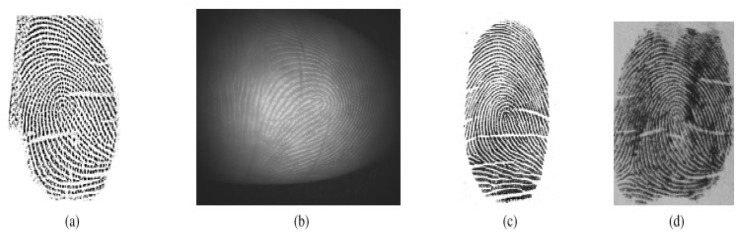
Fingerprints acquired from a single individual using different sensing technologies. (**a**) Touch-based fingerprint using the device Digital Persona (**b**) Touchless fingerprint from digital camera (**c**) Touch-based fingerprint from Futronic (**d**) Rolled fingerprint. Reprinted with permission from Ref. [22], 2018, IEEE.

It is noted that there are some basic differences between contact-based and contactless systems that challenge the matching compatibility of images and lead to performance drop. The former systems acquire gray scale images and have deformation due to the pressure, while the later systems capture RGB images that are mirrored along the vertical axis [25]. Therefore, when handling contactless images, the process of mirroring, conversion of RGB images to grayscale, inversion of background and foreground and estimation of deformation need to be performed before cross-matching. However, there is still a gap for robust deformation correction schemes, and it is yet to be implemented [156]. Figure 17 illustrates the variations of a finger captured from an individual based on contact and contactless nature.

Another issue identified in the literature is DPI alignment for contactless images. Contact-based devices use a metric of spatial dot density and 500 DPI is the ISO/IEC requirement for commercial products [157], while contactless devices do not have a DPI value. Therefore, a normalization process of contactless images to the same size and resolution of the contact print is required. The second constraint is the estimation of ridge frequency. In contactless images, ridge frequency increases towards borders, while it is stable in contact-based prints due to the deformation made by finger pressure on the sensor surface. Lin et al. [22] experimented with a deformation correction model using thin plate splines which resulted in a positive effect on ridge estimation in contactless images.

Table 4 summarizes the existing contact–contactless fingerprint-matching databases and imaging sensors. It is noted that all of the recent work tends to move towards finding resolution on only a sub-domain of the challenges with full effort to obtain contactless-touch-based fingerprint system performance similar to the state-of-the-art touch-based fingerprint systems [36]. There is a lack of study present in the current literature for contact–contactless fingerprint matching. Though few investigations have been carried out these days, the matching process has challenges in different aspects of the contactless fingerprint recognition pipeline such as image capture, image segmentation, pre-processing, feature representation and image matching, etc. [25]. Therefore, the fingerprint recognition in the cross-matching domain is open for researchers, and there is still further investigation needed in the cross-matching biometric domain.

## 5. Challenges and Alternatives

From the overall analysis, a few key challenges are identified in fingerprint systems, which are yet to be resolved. They are (i) fingerprint performance; (ii) environmental factors; (iii) acceptability; and (iv) interoperability. In terms of fingerprint system performance, 2D contactless systems performed less well than contact-based systems. It can only be enhanced by additional sophisticated and special 3D setups with a standard pre-processing [160]. Further, mobile-based commodity devices do not result in a competitive performance yet. Therefore, to achieve a competitive performance, all stages of the contactless fingerprint recognition pipeline should be carefully handled with advanced algorithms and techniques. For instance, image acquisition phases should be carefully monitored with homogeneous illumination, noiseless background, high-quality and speed camera. In a similar way, pre-processing, feature extraction and matching modules were also analyzed to attain the best-performing fingerprint system.

Environmental factors such as illumination variation, very dark and bright and noisy backgrounds, varying camera setup and finger position with the sensor result an extremely negative impact on a system’s performance, particularly during mobile capture. Therefore, robust algorithms for finger detection and segmentation from the (similar skin-color) background are essential. Standard pre-processing and quality assessment techniques should be strictly employed with the system.

Since contactless devices have a higher acceptability compared to contact-based devices, they can be further enhanced by maintaining standard distances between finger and sensor and providing an unconstrained environment. Further, for the cross-matching and interoperability issue, it is observed that a general effort for contact–contactless matching is carried out. However, each module of the fingerprint recognition pipeline should be separately investigated in cross-matching domains.

## 6. Conclusions

This work comprehensively analyzes the rapidly growing contact and contactless fingerprint recognition systems with three significant modules such as sensors, image acquisition and interoperability, and overall challenges are outlined with alternatives. In sensing module, it features a broad spectrum on sensors with recent trends and advances, especially in ultrasonic sensing with CMUTs and PMUTs. Further, recent advances in OCT and its application in 3D-image sensing are discussed. In image acquisition, the challenges of using different contact-based fingerprints such as rolled, plain, latent and live-scan partial are analyzed. Some precautions to prevent performance drops in contact- and contactless-based fingerprint acquisition are outlined. Further, contactless image capture strategies performed on 3D-imaging over 2D-imaging are systematically analyzed. A general effort was performed in the domain of cross-matching in two different directions: (i) cross-matching of fingerprints acquired using contact-based sensors with different techniques and (ii) cross-matching of finger images acquired using contact-based and contactless devices are outlined. Even though experimental methods satisfied the interoperability between contact-based fingerprints captured using different techniques, challenges exist in contact–contactless fingerprint matching, and none of them achieved the accuracy similar to the accuracy of contact–contact matching. Further, 2D contactless schemes have higher acceptability compared to contact-based schemes, while system performance remains a challenge; particularly, mobile-based devices are yet to reach a competitive performance because the feature of portability on mobile devices compromises their performance. More research is yet to be investigated for acquiring robust and interoperable fingerprint systems.

## Figures and Tables

**Table 1:** Image Acquisition Mode.

Mode of Acquisition	Related Issue
Contact acquisition	Ridge structure of the fingerprint in 3D form is eliminated while contacting with the surface. Insufficient and too much pressure can affect the ridge surface.
Flat Fingerprint Acquisition	Finger surface flattened against the surface of the sensor. Different forms and ranges of distortion may occur during the capture
Rolled Fingerprint Acquisition	3D finger structure is converted into a 2D plane by rolling the finger.
Iinked Fingerprint Acquisition	The print impressed on the card is a representation of the ridge surface friction. The digital image acquired from the card by inked impression is a second-order representation of the ridge structure. Errors may occur when converting the inked impression to digital form via optical scanning.
Contactless Acquisition	Optical representation of an Illuminated finger surface is represented in optical where 3D structure is turned into a 2D plane.

**Table 4:** Summary of touch-based and contactless fingerprint datasets used in the literature [36].

Database and Year	Contactless Image Acquisition	Contact-Based Image Acquisition
3D Fingerprint Database-2014 [158]	3D Scanner	CROSSMATCH Verifier 300 LC2.0
Man Tech-2015 [159]	iPhone 4	Cross Match Guardian R2,
PolyU Contactless 2D- Contact 2D Database-2018, [22]	Low-cost camera	URU 4000
Finger Photo and Slap Fingerprint Database-2018 [129]	Smartphone	CrossMatch Guardian 200,
Touchless and Touch-Based Fingerprint Database-2019 [130]	Smartphone	eNBioScan-C1(HFDU08)
ISPFDv2-2020 [155]	Smartphones	Secugen Hamster IV
Finger Photo and Touch-based Fingerprint Database-2021 [36]	Smartphones	URU 4500

## Data Availability

Not applicable.
